# Evaluation of Polyphenolic Compounds Common in Greek Medicinal Plants for Their Antioxidant Effects and Antiviral Activity Against Dengue and Yellow Fever Viruses

**DOI:** 10.3390/antiox14091103

**Published:** 2025-09-10

**Authors:** Eirini Kyriakopoulou, Aliki Tsakni, Evangelos Korakidis, George Mpekoulis, Katerina I. Kalliampakou, Monika Polanska, Jan F. M. Van Impe, Efstathia Tsakali, Dimitra Houhoula, Niki Vassilaki

**Affiliations:** 1 Laboratory of Molecular Virology, Hellenic Pasteur Institute, 11521 Athens, Greece; e.kyriakopoulou@pasteur.gr (E.K.); e.korakidis@pasteur.gr (E.K.); g.mpekoulis@pasteur.gr (G.M.); e.kalliampakou@pasteur.gr (K.I.K.); 2 Department of Food Science and Technology, Faculty of Food Sciences, University of West Attica, 12243 Athens, Greece; atsakni@uniwa.gr (A.T.); etsakali@uniwa.gr (E.T.); dhouhoula@uniwa.gr (D.H.); 3 BioTeC+, Chemical and Biochemical Process Technology and Control, Department of Chemical Engineering, KU Leuven, 9000 Ghent, Belgium; monika.polanska@kuleuven.be (M.P.); jan.vanimpe@kuleuven.be (J.F.M.V.I.)

**Keywords:** polyphenols, Greek medicinal plants, dengue virus, yellow fever virus, antioxidant capacity, DNA scission

## Abstract

Polyphenolic compounds, commonly found in Greek medicinal plants, exhibit promising antiviral and antioxidant properties, making them potential candidates for therapeutic purposes. This study aims to evaluate the antiviral activity of nine selected polyphenols against Dengue virus (DENV) and Yellow Fever virus (YFV) life cycles, alongside their antioxidant capacity determined by the DPPH method and the ABTS assay, and their ability to inhibit DNA strand scission induced by peroxyl radicals. Kaempferol and caffeic acid demonstrated the most potent inhibitory effects on DENV genome replication, while coumaric acid blocked viral entry more effectively. Notably, among the nine compounds, kaempferol exhibited the strongest anti-DENV effect, especially at the level of virus-released infectivity, showing the lowest EC_50_ (3.55 μΜ) and the highest selectivity index (SI = 25.45). In contrast, none of the compounds showed significant antiviral activity against YFV genome replication. Concomitantly, caffeic acid and kaempferol had the highest radical scavenging activity (DPPH and ABTS assays), highlighting their dual properties. Moreover, DNA scission inhibition assays confirmed the strong antioxidant potential of all tested compounds, with caffeic acid and kaempferol achieving the highest inhibition rate of 98.98% and 97.34% respectively. These findings underscore the potential of specific polyphenols, particularly kaempferol and caffeic acid, as antiviral and antioxidant agents targeting DENV and oxidative stress-related damage.

## 1. Introduction

The use of medicinal plants to treat diseases is a practice that dates back to ancient times [1,2]. Recently, the exploration of naturally derived products for their antioxidant and antiviral properties has attracted considerable interest [3,4]. This interest is largely driven by the need for safer and more effective therapeutic alternatives, the rising prevalence of viral infections, and the adoption of healthier lifestyles that encompass the use of natural antioxidant compounds. The beneficial effects of polyphenolic agents, including their antioxidant [5,6,7] and antiviral activities [8,9], have been demonstrated in many studies, establishing a foundation for the development of new pharmaceuticals. It is noteworthy that a large amount of the drugs currently in clinical research and development are either directly or indirectly derived from plants [10].

Greece and its wide range of ecological conditions are home to an exceptional diversity of medicinal and aromatic plants with a significant degree of endemic plants as a result of the country’s geographical and geological features. The Greek medicinal plants have a long history in traditional medicine and other aspects of human life due to their bioactive qualities [11]. The abundance of bioactive compounds in Greek plant extracts, particularly polyphenols such as flavonoids with well-known antioxidant properties, has attracted considerable scientific attention [12]. Oxidative stress contributes significantly to cellular dysfunction and the pathogenesis of many diseases, including viral infections, highlighting the need for natural antioxidants to mitigate the virus-mediated harmful effects while enhancing immune responses [13,14]. Thus, further investigation into the pharmacological properties and therapeutic applications of plant-derived compounds with promising antioxidant and antiviral potential is imperative to advance plant-based drug development [4].

This is of particular significance in the context of highly epidemic viral infections that currently lack approved therapeutic options, such as those caused by *Flaviviridae* viruses, including yellow fever virus (YFV) and dengue virus (DENV). These pathogens continue to pose significant global public health challenges and are known to induce oxidative stress within host cells, underscoring the urgent need for novel therapeutic approaches that can address both their antiviral and antioxidant demands. Elevated levels of reactive oxygen species (ROS) that occur during *Flaviviridae* infection can impair the immune response, damage cellular components, and ultimately contribute to disease severity [15].

DENV infections, although sometimes asymptomatic, often cause high-severity symptoms such as hemorrhagic fever or shock syndrome [16]. Mosquito-borne DENV causes around 400 million infections annually across more than 100 countries [16]. Recently, the spread of mosquito vectors and DENV has extended to numerous countries, including Greece, that had not experienced dengue epidemics in recent years, largely due to climate change [17]. This has raised significant public health concerns, as well as highlighting the country’s vulnerability to future outbreaks of these diseases.

The genome of DENV is a single-stranded positive-sense RNA (+ssRNA) molecule of approximately 10,700 nucleotides, which possesses 5′ and 3′ untranslated regions (UTRs) at both ends of the open reading frame (ORF) that encodes a polyprotein of 3391 amino acids. The process of DENV entry into host cells is initiated by receptor-mediated endocytosis [18]. Then, the viral envelope is fused with the endosomal membrane under acidic conditions [19,20]. This process liberates the viral RNA genome into the cytoplasm, where its translation takes place. Next, the viral polyprotein matures, producing ten distinct structural and nonstructural proteins, which facilitate genome replication and virion assembly [19,20]. The assembly of viral particles occurs on the endoplasmic reticulum (ER) membrane. Then, the immature viral particles travel through the trans-Golgi network (TGN), mature, and are released via exocytosis to infect neighbouring cells [21].

Despite several decades of intensive research, no specific antiviral treatment for the dengue virus has been approved thus far. The limited efficacy of the currently available dengue vaccine underscores the urgent need for new alternative antiviral therapies, particularly in endemic regions, to reduce disease severity and associated mortality. It has been demonstrated that polyphenolic compounds obtained from plant extracts possess the capacity to exhibit anti-flaviviral activity [22,23]. Extracts from the plants *Ocimum basilicum* L., *Mentha piperita* L., *Mentha spicata* L., and *Aloysia citrodora* have been shown to exert free radical scavenging, antimicrobial activity against pathogenic bacteria, and antiviral effects [24,25,26,27]. More specifically, in Greece, *Ocimum basilicum* L. has been traditionally used for headaches, bronchitis, insomnia, and as an antipyretic [28,29]. It has also shown antibacterial and antifungal activity similar to *Mentha spicata* L. [30]. *Mentha spicata* L. has also been used for hypertension, gastrointestinal and cardiovascular issues, while other *Mentha* spp. have been used for digestive and respiratory problems [28,29]. *Mentha piperita* L. and *Aloysia citrodora* are traditionally used as spasmolytics, with the latter also employed for gastrointestinal disorders [29]. All four plant species have also been traditionally used to relieve common cold symptoms, hinting at their antiviral potential [29,31]. In this study, to gain a deeper knowledge of their properties, we evaluated the antioxidant capacity and antiviral activities of specific polyphenolic compounds identified in these plants. Specifically, regarding their anti-flaviviral function, we studied their effects on viral entry, replication, and release infectivity, with the ambition of developing potential antiviral drugs.

## 2. Materials and Methods

### 2.1. Standards

Commercially available phenolic compounds, including ferulic acid, quercetin, rosmarinic acid, naringenin, coumaric acid, rutin, luteolin, caffeic acid, and kaempferol, were acquired from DR EHRENSTORFER GmbH (Augsburg, Germany). DR EHRENSTORFER GmbH additionally provided supercoiled pBR322 DNA, 2,2-diphenyl-1-picrylhydrazyl (DPPH•) and 2,2′-azinobis-(3-ethylbenzothiazoline-6-sulfonic acid) diammonium salt (ABTS).

### 2.2. Methodology for Sample Preparation and Extraction

Plant materials were sampled from the area of Rethymno (Crete, Greece) at an altitude of approximately 200 m (182–228 m) throughout June 2021. The species included *Ocimum basilicum* L., *Mentha piperita* L., and *Mentha spicata* L., all belonging to the *Lamiaceae* family, and *Aloysia citrodora*, which belongs to the *Verbenaceae* family. The plant materials were identified based on morphological characteristics at the University of West Attica (Athens, Greece). The leaves of plant material were air-dried at ambient room temperature (approximately 25 °C) in the shade for one week until constant weight was achieved, in order to prevent degradation of thermolabile compounds. The dried leaf material was pulverized using a mechanical grinder. For each species, 10 g of the resulting powder was transferred into conical flasks of 100 mL capacity. Extraction was carried out by maceration under ambient conditions for half a month to obtain a high concentration of phenolic and antioxidant compounds, with intermittent stirring, employing a 10:90 ethanol–water solvent system [32]. Filtration and centrifugation (8000 revolutions per minute for 15 min) were performed on the derived extracts. Solvent removal was accomplished under reduced atmospheric pressure using a rotary evaporator (50 °C for 2 h). After dissolving in methanol, the remaining material was filtered using a 0.22 μm polyvinylidene difluoride membrane and prepared for chromatographic analysis. The extracts were maintained at 2 °C to preserve the stability of thermolabile bioactive molecules.

### 2.3. Preparation of Standard Solutions of Polyphenolic Compounds

#### 2.3.1. Preparation of Standard Solutions of Polyphenolic Compounds for HPLC Analysis

The phenolic compounds were dissolved in methanol to prepare stock solutions. Five different concentrations (100, 50, 20, 10, 5 μg/mL) were prepared for each phenolic compound and used as standards in order to construct the calibration curves. In the calibration curves, the y-axis represents the absorption of each dilution of the standard, while the x-axis represents the concentrations of the known standard solutions. These calibration curves were used for the quantitative determination of phenolic compounds in the plant extracts.

#### 2.3.2. Preparation of Standard Solutions of Polyphenolic Compounds for Biological Assays

For the evaluation of antioxidant activity and the assessment of their protective effect against peroxyl radical-induced DNA damage, the phenolic compounds were dissolved in methanol. For antiviral activity and cytotoxicity assessment, the compounds were dissolved in dimethyl sulfoxide (DMSO). All test solutions were freshly prepared prior to the experiments and appropriately diluted to the desired concentrations.

### 2.4. Liquid Chromatography of High Performance (HPLC) and Diode Array Detection

The natural phenolic compounds were identified in plant extracts using HPLC coupled with a Diode Array Detector (HPLC-DAD) (VWR^®^ Hitachi Elite LaChrom system, VWR, Darmstadt, Germany). The chromatographic separation was carried out using a column that retains hydrophobic molecules—SVEA C18 with dimensions 150 mm × 4.6 mm and 5 μm particle size (Nanologica, Stockholm, Sweden)—under a gradient elution system. The column operated using a constant flow rate of 0.5 mL/min at 30 °C. The mobile phase consisted of three solvent systems: A) water containing 1% formic acid, B) methanol containing 1% formic acid, and C) acetonitrile containing 1% formic acid. The column running program was applied as follow: 90% A, 6% B, 4% C from 0 to 5 min, adjusted to 85% A, 9% B and 6% C from 5 to 30 min, between 30 and 60 min the composition changed to 71% A, 17.4% B and 11.6% C, 0% A, 85% B and 15% C from 60 to 63 min. Finally, between 63 and 65 min, the system was re-equilibrated to 90% A, 6% B, and 4% C [12]. A volume of 20 μL was used for injection, and the detection was conducted at a wavelength of 280 nm—a wavelength frequently used for the analysis of phenolic compounds due to their strong absorbance in this region. The identification of the bioactive compounds in the extracts was carried out using their retention times and λmax (qualitative analysis) and the construction of standard calibration curves (quantitative determination).

### 2.5. Cell Culture, Viral Stocks, and Cell Infection

#### 2.5.1. Cells and Viral Constructs

Huh7 [33] and Huh7-YF cells [34] were maintained at 37 °C and 5% CO_2_, in Dulbecco’s modified Eagle’s medium (DMEM) of high glucose (4.5 g/L) supplemented with 10% (*v*/*v*) heat-inactivated fetal bovine serum (FBS), 2 mM L-glutamine, 0.1 mM non-essential amino acids, and antibiotics 100 U/mL penicillin, 100 μg/mL streptomycin, henceforth marked as complete DMEM. In the case of Huh7-YF cells, 0.5 μg/mL puromycin was added to the complete DMEM. The plasmid carrying the full-length DENV genomic sequence pFK-DVR2A (co-expressing the reporter gene Renilla luciferase), derived from the DENV-2 16681 strain, has been described elsewhere [35,36]. The Huh7-YF cell line carries the pYF-hRUPac subgenomic replicon of YFV-17D [37], which co-expresses the Renilla luciferase gene, the activity of which is indicative of yellow fever virus (YFV) replication.

#### 2.5.2. DENV Stocks and Cell Infection

The plasmid pFK-DVR2A was XbaI-linearized prior to in vitro transcription, as described elsewhere [35]. The concentration and quality of the transcribed RNA were determined using a Nanodrop spectrophotometer, while its integrity was verified by electrophoresis using a denaturing agarose gel. The in vitro transcription-generated full-length DENV RNA was introduced via electroporation into VeroE6 cells [38]. After twenty-four hours, the culture medium was replaced with complete DMEM supplemented with 15 mM HEPES (pH 7.5), and the incubation of cells was continued until the onset of the DENV infection-induced cytopathic effect (CPE). Supernatants were collected from the electroporated cells starting from day 4 until day 7 post-transfection, pooled, filtered through a 0.45 μm filter, aliquoted, and kept at −80 °C. These supernatants were passaged to new VeroE6 cells to produce DENV stocks. The titer of the viral stock was determined using a standard plaque assay following inoculation of VeroE6 cells for 4 h [39].

For cell culture infection experiments, the produced DENV stocks were used for cell inoculation (4 h), then complete DMEM was used to replace the inoculum, and the cells were further incubated and treated as referred in the experimental procedure each time.

### 2.6. Determination of Cytotoxicity

The putative cytotoxicity of the phenolic compounds was evaluated by measuring the intracellular ATP levels of Huh7 cells incubated with serial dilutions of each compound. In brief, in 96-well plates, 10^4^ cells per well were seeded and treated at 24 h post-seeding with different concentrations (500, 250, 125 μΜ) of the compounds diluted in dimethyl sulfoxide (DMSO) and incubated for 72 h. Cells treated with only DMSO were used as the control. To determine the intracellular ATP, the ViaLight plus kit (Lonza, Basel, Switzerland) was used according to the manufacturer’s instructions. The cells were lysed at 72 h of incubation, and the luminescence was measured for 1 s using the GloMax 20/20 single-tube luminometer (Promega Corporation, Madison, WI, USA). For each compound, the concentration that caused 50% cell death (CC_50_) was determined based on the difference in intracellular ATP levels observed between the compound-treated and the DMSO-treated (control) cells. Drug concentrations were converted to log-X format, and non-linear regression analysis was used to determine the CC50 values using Prism 6.0 software (GraphPad Software Inc., San Diego, CA, USA).

### 2.7. Cell-Based Antiviral Assays

First, the activity of the natural compounds against DENV was assessed for viral entry, genome replication, and released infectivity. To examine effects on the virus entry, cells were preincubated, 24 h after seeding, with increasing concentrations of the compounds or the solvent DMSO in complete DMEM for 2 h. Then, the culture medium was changed, and the cells were inoculated with DENV virions at a multiplicity of infection (MOI) of 0.01, in the presence of the corresponding compound. To detect effects on viral replication, Huh7 cells were inoculated with DENV virions (MOI = 0.01) for 4 h in the absence of the compounds, then the medium was replaced with complete DMEM containing the respective compound or DMSO (control). To evaluate effects on the DENV released infectivity, supernatants were collected from cells to which the compound had been added after virus inoculation and used to infect new cells plated at 30% density on a 96-well plate. The cells were incubated with the supernatants containing the virus for 16 h, and then the culture medium was changed to complete DMEM medium. After 72 h, the cells were lysed, and the Renilla luciferase activity (R-Luc) was determined.

In the case of YFV, Huh7 cells containing the replicon of the virus were used (Huh7-YF). The Huh7-YF cells were seeded at a concentration of 10^4^ cells per well of a 96-well plate, in 200 μL complete DMEM containing puromycin. After 24 h incubation, the culture medium was replaced with 100 μL of new puromycin-free complete DMEM supplemented with the phenolic compound. Three serial dilutions of each compound were used. Following a 72-h incubation, the cells were lysed, and the activity of Renilla luciferase (R-Luc) was determined.

### 2.8. Renilla Luciferase (R-Luc) and Bradford Assays

The concentration of proteins in the cell lysates was determined using the Bradford assay reagent (Bio-Rad, Hercules, CA, USA).

The R-Luc activity was evaluated in cell lysates using a coelenterazine-containing buffer: 12 μM coelenterazine (Promega) in 500 mM NaCl, 50 mM potassium phosphate (pH 7.4), 1 mM EDTA. Luminescence was recorded using the single-tube luminometer GloMax 20/20 (Promega Corporation, Madison, WI, USA) over a period of 10 s. The activity of Renilla luciferase obtained was then normalised to the total protein concentration. R-Luc activity was expressed as a percentage of luminescence units (RLU) relative to the corresponding mean value (set as 100%) obtained from the DMSO-treated (control) cells. For each compound, the concentration that results in a 50% diminution of the Renilla luciferase activity corresponds to the half-maximal effective concentration (EC_50_) and was determined by non-linear regression analysis, after conversion of drug concentrations to logX using Prism 243 5.0 software (GraphPad Software Inc.).

### 2.9. Antioxidant Capacity

#### 2.9.1. DPPH Method for Measuring Radical Scavenging Activity

The deep purple-coloured 2,2-diphenyl-1-picrylhydrazyl (DPPH) is often used to assess antioxidant capability [40]. DPPH radicals can be easily neutralized by phenolic compounds, either by donating hydrogen atoms or electrons. A freshly prepared methanol solution of 10^−6^ M DPPH was used [41]. A volume of 3400 μL of DPPH solution was mixed with a volume of 100 μL of methanol containing the phenolic compound. Different concentrations (3.44, 6.88, 13.75, 27.50, 55.00 μg/mL) of phenolic compounds were used. After 45 min of incubation in the dark at room temperature, we measured the absorbance at 517 nm (A_sample_) using a VIS spectrophotometer (Thermo Spectronic Helios Epsilon, Waltham, MA, USA). For the control, we used 100 μL of methanol instead of the solution of the compound, and its absorbance (A_control_) was recorded [24]. The equation below was applied to calculate radical scavenging activity:

$$\%\;\mathrm{Radical}\;\mathrm{Scavenging}\;\mathrm{Activity}\;=\;\frac{Acontrol-Asample}{Acontrol}*100\%$$

The concentration of the compound that results in a 50% reduction in radicals corresponds to the half-maximal inhibitory concentration (IC_50_), and it was determined by non-linear regression analysis.

#### 2.9.2. ABTS Method for Measuring Radical Scavenging Activity

The 2,2′-azinobis (3-ethylbenzothiazoline-6-sulfonic acid) diammonium salt (ABTS) radical cation decolorization assay is a commonly used spectrophotometric technique for evaluating the antioxidant capacity of different natural compounds. In the presence of antioxidant substances, the radical ABTS^+•^ discolors from its intense blue-green colour. The radical was produced by combining equal volumes of 7 mM ABTS (38.4 mg ABTS in 10 mL H_2_O) and 2.45 mM potassium persulfate stock solution (6.62 mg K_2_S_2_O_8_ in 10 mL H_2_O), followed by incubation in the dark at room temperature for 12 h. The prepared solution was subsequently subjected to dilution with ethanol until its absorbance was adjusted to 0.700 at 734 nm, using a VIS spectrophotometer (Thermo Spectronic Helios Epsilon, Waltham, MA, USA). Ethanol (100 μL) was measured as a control sample (A_control_). An aliquot of 3400 μL of the diluted ABTS^+•^ solution was combined with 100 μL of different concentrations of the phenolic compounds (3.44, 6.88, 13.75, 27.50, 55.00 μg/mL). The mixture was incubated at 30 °C for 6 min, and the absorbance was thereafter recorded at 734 nm (A_sample_) [42]. The ABTS^+•^ inhibition percentage was determined using the following equation:

$$\%\;\mathrm{Radical}\;\mathrm{Scavenging}\;\mathrm{Activity}\;=\;\frac{Acontrol-Asample}{Acontrol}*100\%$$

### 2.10. DNA Strand Break Assay

The efficacy of the tested phenolic compounds to inhibit the peroxyl radicals-induced cleavage of the DNA strand was measured using a supercoiled DNA according to the method of Albishi et al. [43], with some modifications. A solution of pBR322 supercoiled plasmid DNA (derived from *Escherichia coli* RRI) at a concentration of 50 μg/mL in 0.5 M phosphate-buffered saline (PBS), and a solution of 2,2′-azobis (2-amidinopropane) hydrochloride (AAPH), a free radical generator, were used. A mixture containing 4 μL of the solution of the pBR322 supercoiled plasmid DNA, 4 μL of AAPH 30 mM, 2 μL of PBS, and 2 μL of the phenolic compounds (at IC_50_ concentrations calculated by DPPH method) was incubated for 20 min at 37 °C. For the blank, a mix lacking the sample was used. For the control, a mix containing neither AAPH nor the sample was included in the experiment. At the end of incubation, each sample was analysed through electrophoresis (100 V for 2 h) in a 0.8% agarose gel to assess DNA integrity, and the DNA bands were observed under ultraviolet light [44]. Band analysis for DNA scission quantification was performed using the system MiniBIS Pro (DNR Bio-Imaging Systems Ltd., Neve Yamin, Israel) and presented as a percentage.

$$\%\;\mathrm{DNA}\;\mathrm{preservation}=\frac{Band\;intensity\;of\;supercoiled\;DNA\;with\;AAPH\;and\;phenolic\;compound\;}{Band\;intensity\;of\;supercoiled\;DNA\;in\;the\;control\;sample}\;*100\%$$

### 2.11. Statistical Analysis

We used the GraphPad Prism software (version 6.0) to calculate CC_50_, EC_50,_ and IC_50_ values. Student’s *t*-test was used to compare cytotoxicity and antiviral activity values for each treatment condition with values obtained from DMSO-treated (control) cells, considering a *p*-value < 0.05 as statistically significant.

## 3. Results

### 3.1. Separation and Analysis of Greek Medicinal Plant Extracts

#### 3.1.1. Characterization of Plants’ Extracts by High Performance Liquid Chromatography—Diode Array Detection

**Figure 1:**
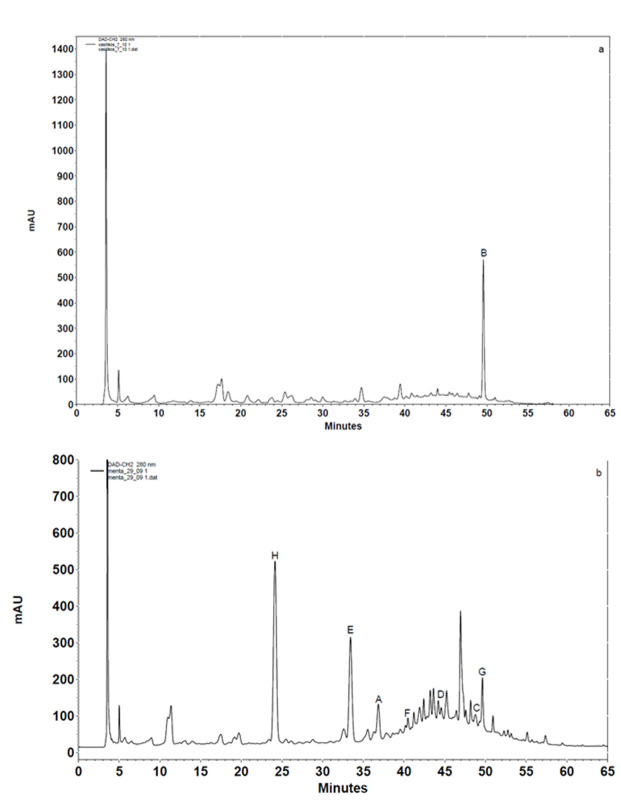

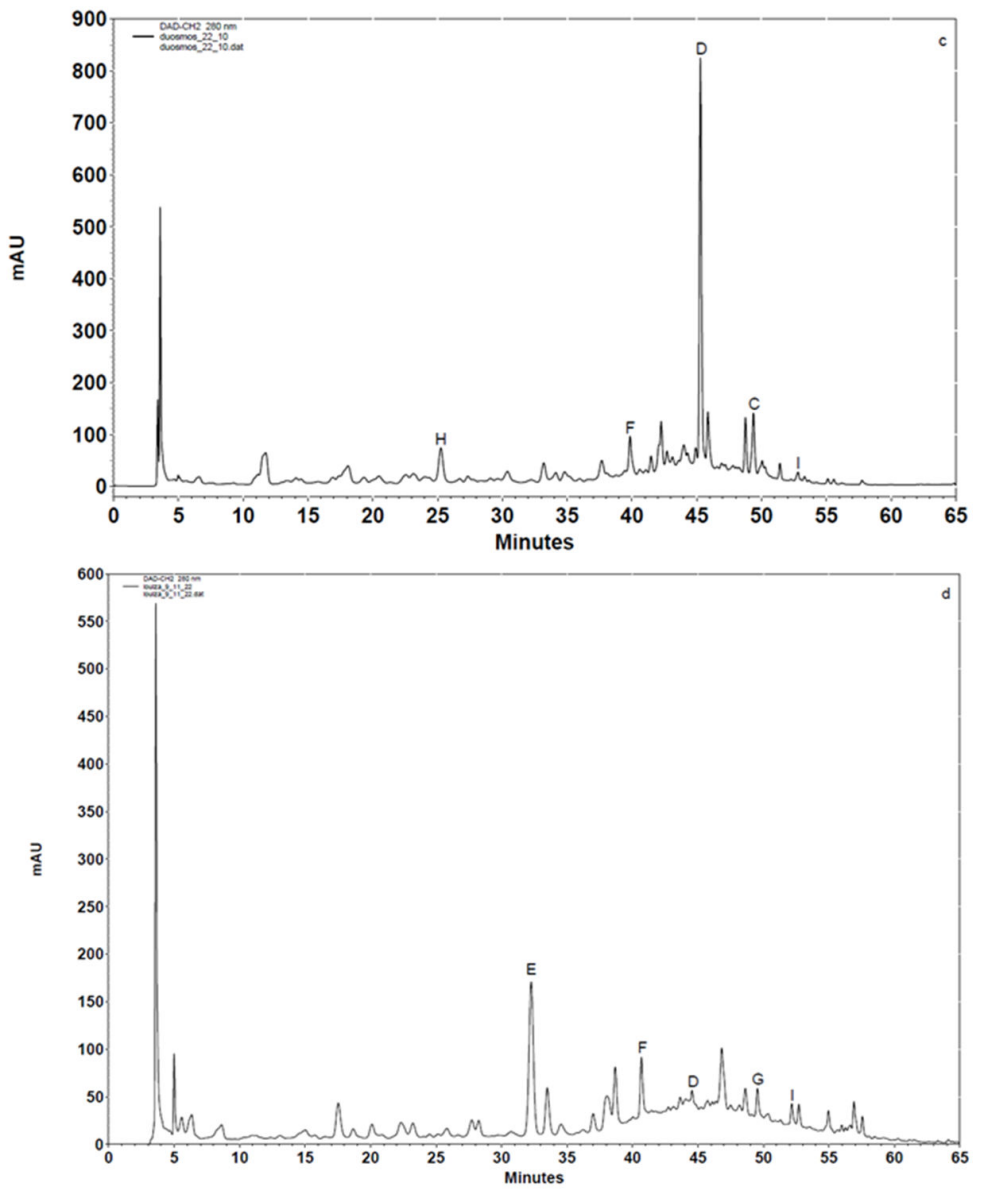
Chromatographic analysis of four aromatic natural herbs: (**a**) *Ocimum basilicum* L., (**b**) *Mentha piperita* L., (**c**) *Mentha spicata* L., and (**d**) *Aloysia citrodora*. In the panels, the peak of the spectrum of each compound is mentioned: (A) ferulic acid, (B) naringenin, (C) quercetin, (D) rosmarinic acid, (E) coumaric acid, (F) rutin, (G) luteolin, (H) caffeic acid and (I) kaempferol, and the respective retention time is depicted.

Nine polyphenolic compounds (flavonoids and hydroxycinnamic acids) were identified in the following species of the Greek flora: *Ocimum basilicum* L., *Mentha piperita* L., *Mentha spicata* L., and *Aloysia citrodora* (Table 1, Figure 1), using HPLC-DAD.

The residual plant materials, weighing 800 μg, after solvent removal by rotary evaporation, were diluted in 1 mL of methanol. The phenolic content was determined in this solution. Rosmarinic acid showed the highest concentration in *Mentha spicata* (126.38 μg/mL), while it was also detected in *Mentha piperita* and *Aloysia citrodora* (Figure 1b–d). Caffeic acid was found mainly in *Mentha piperita* (116.89 μg/mL), but also in *Mentha spicata* (Figure 1b,c). Quercetin, a potent flavonoid, was detected with a maximum concentration of 46.03 μg/mL in *Mentha spicata* (Figure 1c). Rutin levels ranged from 40.58 μg/mL in *Mentha spicata* to 8.32 μg/mL in *Mentha piperita* (Figure 1b–d). Coumaric acid was predominantly detected in *Mentha piperita* at a concentration of 32.21 μg/mL, with a lower amount observed in *Aloysia citrodora* (18.66 μg/mL) (Figure 1b,d). Similarly, luteolin was found in *Mentha piperita* at 27.64 μg/mL and in *Aloysia citrodora* at a reduced concentration of 7.79 μg/mL (Figure 1b,d). Naringenin was notably abundant in *Ocimum basilicum*, reaching a concentration of 69.00 μg/mL (Figure 1a), whereas kaempferol was exclusively detected in *Mentha spicata* and *Aloysia citrodora*. Overall, the findings confirm the presence of bioactive phenolic compounds in the natural extracts, as indicated by the absorption maxima values in the UV spectra of the relevant compounds. The results are consistent with previously published studies [45,46,47].

In addition to the values expressed as μg/mL of the extract’s solutions, the concentrations of the identified polyphenols were also expressed as μg/g dry plant weight, following the extraction conditions (10 g of dry plant material extracted and redissolved in 1 mL of methanol). Naringenin was quantified in *Ocimum basilicum* extract at 6.9 μg/g dry weight. Similarly, in *Mentha piperita,* ferulic acid was 3.19 μg/g, quercetin 0.63 μg/g, rosmarinic acid 0.60 μg/g, coumaric acid 3.22 μg/g, rutin 0.83 μg/g, luteolin 2.76 μg/g, and caffeic acid 11.69 μg/g. In *Mentha spicata,* the detected concentrations were 4.60 μg/g for quercetin, 12.64 μg/g for rosmarinic acid, 4.06 μg/g for rutin, 1.95 μg/g for caffeic acid, and 0.74 μg/g for kaempferol. In *Aloysia citrodora*, quantified levels were: 0.48 μg/g rosmarinic acid, 1.87 μg/g coumaric acid, 3.31 μg/g rutin, 0.78 μg/g luteolin, and 0.41 μg/g kaempferol.

The determination of the phytochemical constituents in the plant extracts was conducted through two approaches: (1) the incorporation of internal standards to enhance analytical accuracy, (2) comparative analysis of the retention times and absorption maxima values of the polyphenolic compounds present in the extracts against those of the corresponding external standards.

#### 3.1.2. Construction of Reference Calibration Curves

Identification of phenolic compounds in the plant extracts was performed by comparison with standard solutions. Specifically, a comparative analysis of retention time and maximum absorbance wavelength (λmax) was undertaken for compound verification (Table S1). The reference calibration curves for ferulic acid, quercetin, rosmarinic acid, naringenin, coumaric acid, luteolin, caffeic acid, and kaempferol are illustrated in Figure S1. Chromatograms of both the nine bioactive compounds and the extracts were recorded at 280 nm, a wavelength chosen for its efficient separation capability. Therefore, the concentration of each compound in the plant extracts was quantified using standard calibration curves.

The most abundant natural constituents identified within the studied extracts were the phenolic compounds described in Section 3.1.1. Therefore, commercially available standards of these compounds were used to evaluate their biological properties. The use of pure reference compounds was preferred to avoid the interference that usually arises from the chemical complexity of crude extracts. The identification of these compounds in various plant species, commonly in the Mediterranean diet, further strengthens the link between the biological activities of pure phenolic standards and their nutritional and medicinal value. Specifically, the bioactive properties being investigated include their antiviral and antioxidant potential, which is evaluated using various in vitro methodologies.

### 3.2. Cytotoxicity Assessment of Polyphenolic Compounds

Prior to investigating the antiviral properties of the nine polyphenols, we evaluated their cytotoxicity. Their effect on cell viability was determined by measuring intracellular ATP levels. Prior to use in cell culture, polyphenols were diluted in DMSO. The Huh7 cells were incubated with increasing concentrations (125, 250, 500 μΜ) of these compounds or were mock-treated with DMSO (control cells) for 72 h, and subsequently, the cells were lysed, and the intracellular ATP was measured. For all natural compounds, the half maximal cytotoxic concentration (CC_50_) corresponding to a 50% reduction in cell viability was determined and is shown in Table 2. The corresponding CC_50_ values, expressed in μg/mL, are provided in Table S2. Most compounds did not exhibit cytotoxicity at the concentrations used; their CC_50_ values were above 500 μM, except for naringenin and kaempferol, which had CC_50_ values of 373 μM and 90.36 μM, respectively.

### 3.3. Evaluation of Effects of Phenolic Compounds Against the Life Cycle Stages of DENV

To screen the plant polyphenols against DENV, we used cell culture-produced viral stocks of the full-length reporter DENV (DVR2A) that co-expresses the Renilla luciferase (R-Luc) gene. As the expression of R-Luc is linked with the expression of viral proteins, the activity of R-Luc is representative of the viral replication. The aim was to study the effect of the compounds on the virus life cycle and specifically on the stages of viral entry, genome replication, and released infectivity, which may be potential targets. For this purpose, Huh7 cells, preincubated or not with the compounds, were infected with DVR2A at MOI = 0.01 pfu/cell, in the presence or absence of the corresponding compound. The compounds were used in non-cytotoxic concentrations, based on their respective CC_50_ values (Table 2).

As compared to DMSO-treated DENV-infected cells (control cells), differences in R-Luc activity of cells that were preincubated with the compound reflect the compound’s effect on both viral entry and genome replication, whereas differences in R-Luc activity of cells that were incubated with the compound after the virus inoculum was withdrawn correspond to effects on viral replication. By comparing the two conditions, it is determined whether a compound influences the entry of the viral particles into the cell. To evaluate the effect of the compounds against virus particle release, supernatants of infected Huh7 cells that were incubated with the compound after the virus inoculum was withdrawn, were collected at 72 h post-treatment and used for the infection of naive Huh7 cells (2nd cycle of infection). After 4 h of incubation, the cell culture medium was changed with complete DMEM, and the cells were incubated for 72 h prior to lysis. This allowed us to determine possible effects on the virus’s released infectivity. Then, the half maximal effective concentration EC_50_ values for the viral entry, replication, and released infectivity were calculated for each compound by non-linear regression analysis derived from luciferase values, and the ratio of CC_50_ to EC_50_ was used to calculate the selectivity index (SI) of the compound (Table 3 and Table S2).

**Figure 2:**
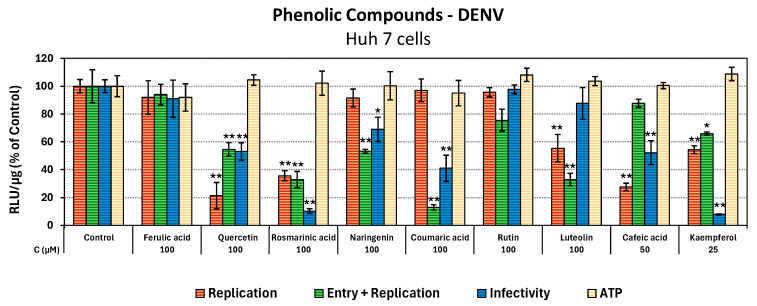
Evaluation of nine phenolic compounds on the life stages of DENV and on cell viability. Huh7 cells were infected with DVR2A, either with preincubation (entry + replication) or without (replication) of the compounds. The cells were then incubated for 72 h with the compounds at the indicated concentrations. The supernatants of the cells in which the compounds were added after the virus inoculum was withdrawn were collected and used to infect new Huh7 cells to evaluate virus infectivity. To evaluate the effect of the compound on cell viability, non-infected Huh7 cells were incubated with the corresponding concentrations of the compound. R-Luc activity and intracellular ATP levels were measured as indicators of viral replication and cell viability, respectively, and then they were normalised to total protein (RLU/μg total protein). The mean values of the infected-DMSO-treated cells for viral replication and the non-infected DMSO-treated cells for cell viability were set to one hundred (control). Bars depict mean values from three experiments performed in triplicate, and error bars stand for standard deviation. * *p* < 0.05, ** *p* < 0.01 vs. control, based on Student’s *t*-test.

As demonstrated in Figure 2, among the natural substances tested, kaempferol and caffeic acid exhibited the strongest activity against DENV genome replication, followed by quercetin and rosmarinic acid, with EC_50_ values of 31.48 μΜ, 35.87 μΜ, 58.46 μΜ, and 76.18 μΜ, respectively. The remaining phytochemical substances demonstrated no inhibitory impact on viral replication. Although coumaric acid was ineffective against viral replication, it emerged as the most efficacious compound against viral entry, exhibiting an EC_50_ of 47.99 μΜ, followed by luteolin with an EC_50_ value of 72.65 μΜ, indicating at least a partial effect on viral entry, compared to an EC_50_ of 107.3 μM concerning viral replication. Regarding DENV released infectivity, kaempferol showed superior antiviral efficacy, as evidenced by its lowest EC_50_ value of 3.55 μM and a high SI of 25.45, emerging as the most promising and potent compound. Furthermore, rosmarinic and caffeic acid also exhibited antiviral activity in terms of DENV released infectivity, with EC_50_ values of 37.12 and 51.49 μΜ, respectively. Conversely, ferulic acid, narigenin, and rutin exhibited no antiviral properties or EC_50_ > 100 μΜ against the tested stages of the viral life cycle. Overall, the results highlight kaempferol as the most potent and promising inhibitor of DENV genome replication and released infectivity, with notable contributions from caffeic acid and rosmarinic acid at the same life stages, while coumaric acid emerged as the most effective compound against viral entry.

**Figure 3:**
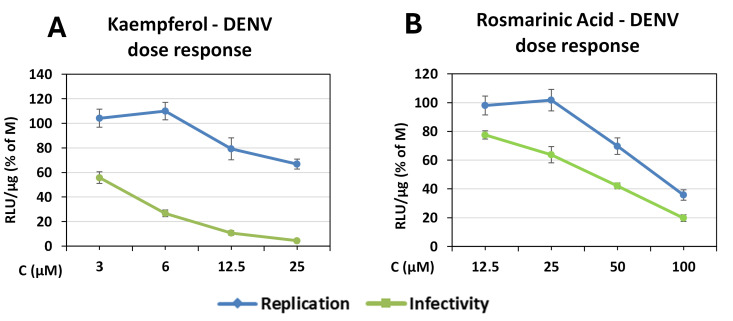
Dose–response curves for kaempferol and rosmarinic acid against DENV. Huh7 cells were infected with DVR2A and then incubated for 72 h with the compounds at the serial dilutions mentioned. The supernatants were collected and used to infect new Huh7 cells (infectivity evaluation). Viral replication (R-Luc) was measured (RLU/μg of protein) and depicted as a percentage of the values from the DMSO-treated cells (control). The points represent mean values from three independent experiments carried out in triplicate, and the error bars represent standard deviation.

Due to the superior efficacy of kaempferol in inhibiting both DENV genome replication and released infectivity, as well as the antiviral activity of rosmarinic acid against both of these stages of the viral life cycle, we generated dose–response curves to characterize their antiviral activity in greater detail (Figure 3). Based on EC_50_ values shown above, we selected for kaempferol the concentrations of 25, 12.5, 6, and 3 μM to be depicted, while for rosmarinic acid, the concentrations of 100, 50, 25, and 12.5 μM were shown.

### 3.4. Evaluation of Phenolic Compounds for Inhibitory Effects on YFV Replication

**Figure 4:**
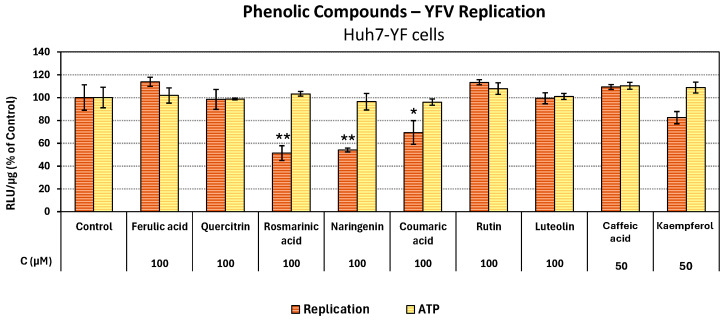
Evaluation of natural compounds against YFV replication and on cell viability. Huh7-YF cells, harboring the YFV-17D replicon, were incubated for 72 h with different concentrations of the compounds. R-Luc activity, an indicator of viral replication, and intracellular ATP levels, an indicator of cell viability, were normalised to total protein (RLU/μg total protein). The mean values of the infected-DMSO-treated cells for viral replication and the non-infected DMSO-treated cells for cell viability were set to one hundred (control). Bars depict the mean values of three independent experiments carried out in triplicate, and error bars depict the standard deviation. * *p* < 0.05, ** *p* < 0.01 compared to control (Student’s *t*-test).

To investigate whether the studied polyphenols exhibit broad-spectrum antiviral activity against the *Flavivirus* genus, we also evaluated their efficacy against Yellow Fever Virus (YFV). In order to evaluate the compounds for their antiviral activity against YFV, the Huh7-YF cell line was employed, which expresses the subgenomic replicon of YFV-17D. Initially, the cells were subjected to incubation with the compounds at serial concentrations for 72 h. The quantification of viral replication was assessed by measuring Renilla luciferase activity, which is co-expressed with the viral replicon and corresponds to the levels of viral replication. The EC_50_ values and their respective SI were calculated and presented in Table 4. As shown in Figure 4, most compounds did not exhibit any effect against YFV replication, except for rosmarinic acid and naringenin, which showed a slight effect with EC_50_ values estimated at 98.03 μΜ and 101.70 μΜ, respectively (Table 4).

### 3.5. Evaluation of the Antioxidant Activity of the Phenolic Substances

#### 3.5.1. DPPH Method for Measuring Radical Scavenging Activity

Polyphenols’ antioxidant activity is regarded as perhaps their most prominent and beneficial property. Much of the diverse biological impact of these compounds on human health stems from their ability to counteract oxidative stress [48]. The antioxidant effectiveness of the compounds under investigation was assessed using the DPPH method. IC_50_ values (μg/mL) were used to represent the DPPH assay data. IC_50_ values correspond to the sample concentration required to achieve a 50% reduction in absorbance at 517 nm [49].

**Figure 5:**
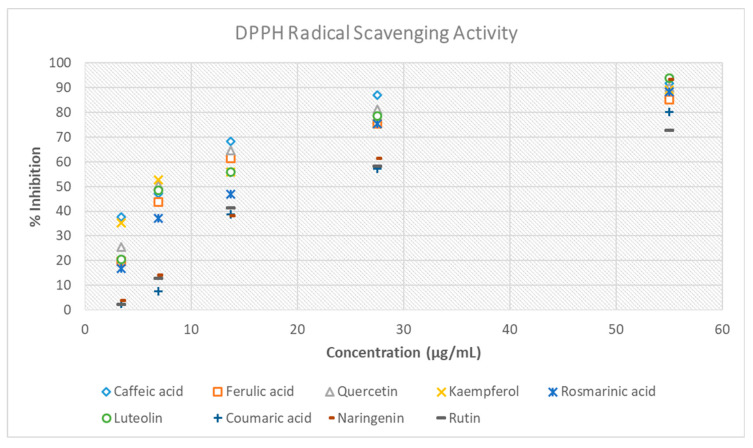
Antioxidant potential of the nine polyphenols against DPPH radicals.

The IC_50_ values of the compounds, caffeic acid, ferulic acid, quercetin, kaempferol, rosmarinic acid, luteolin, coumaric acid, naringenin, and rutin were 6.37, 10.14, 8.05, 7.40, 12.28, 9.48, 21.17, 17.45, and 21.86 μg/mL, respectively (Table 5, Figure 5). These results indicate that caffeic acid exhibited the strongest antioxidant activity among the tested polyphenols, with the remaining compounds displaying progressively lower activity in the following order: kaempferol, quercetin, luteolin, ferulic acid, rosmarinic acid, naringenin, coumaric acid, and rutin. Caffeic acid and kaempferol exhibit the highest antioxidant activity, thereby suggesting a potential mechanism by which they inhibit DENV replication through the mitigation of oxidative stress essential for viral propagation [15]. Nevertheless, no appreciable differences were observed between the majority of the samples. Within the group of the examined compounds, rutin exhibited the weakest antioxidant activity, reflected by its highest IC_50_ value (21.86 μg/mL).

#### 3.5.2. ABTS Method for Measuring Radical Scavenging Activity

The antioxidant capacity of the nine polyphenols was quantified using the ABTS radical cation decolorization assay, and their radical scavenging efficacy was characterized based on their IC_50_ values [50].

**Figure 6:**
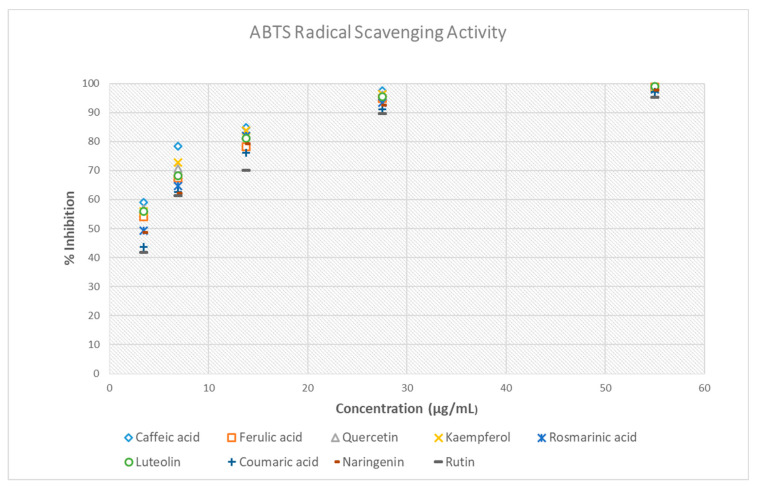
Antioxidant potential of the nine polyphenols determined by ABTS assay.

The IC_50_ values for caffeic acid, ferulic acid, quercetin, kaempferol, rosmarinic acid, luteolin, coumaric acid, naringenin, and rutin were found to be 1.28, 2.51, 1.93, 1.84, 3.05, 2.20, 4.00, 3.37, and 4.54 μg/mL, respectively (Table 6, Figure 6). Among all the studied compounds, caffeic acid exhibited the highest activity, as evidenced by its lowest IC_50_ value, reflecting a high efficiency in scavenging ABTS^+^ radicals. Kaempferol and quercetin were shown to have strong antioxidant properties, similar to caffeic acid. Luteolin and ferulic acid exhibited slightly higher IC_50_ values, indicating a slight decrease in radical-scavenging efficiency compared to the three most potent compounds. Coumaric acid and rutin had the lowest activity among the substances examined, indicating comparatively lower efficiency in neutralizing radicals. Overall, the findings indicate that the antioxidant activity of the polyphenols is influenced by the number and the position of hydroxyl groups and the presence of specific structural features, like catechol moieties on the aromatic ring [51]. These findings underscore that certain polyphenols, especially caffeic acid, kaempferol, and quercetin, may act as effective natural antioxidants, potentially decreasing oxidative stress-induced damage in biological systems.

Thus, the results of the ABTS assay are in agreement with those of the DPPH method, concerning the order of the antioxidant potential of the tested compounds, as the strongest activity was observed in the case of caffeic acid, followed by that of kaemferol, quercetin, luteolin, ferulic acid, rosmarinic acid, naringenin, coumaric acid, and finally of rutin.

### 3.6. DNA Strand Break Assay

**Figure 7:**
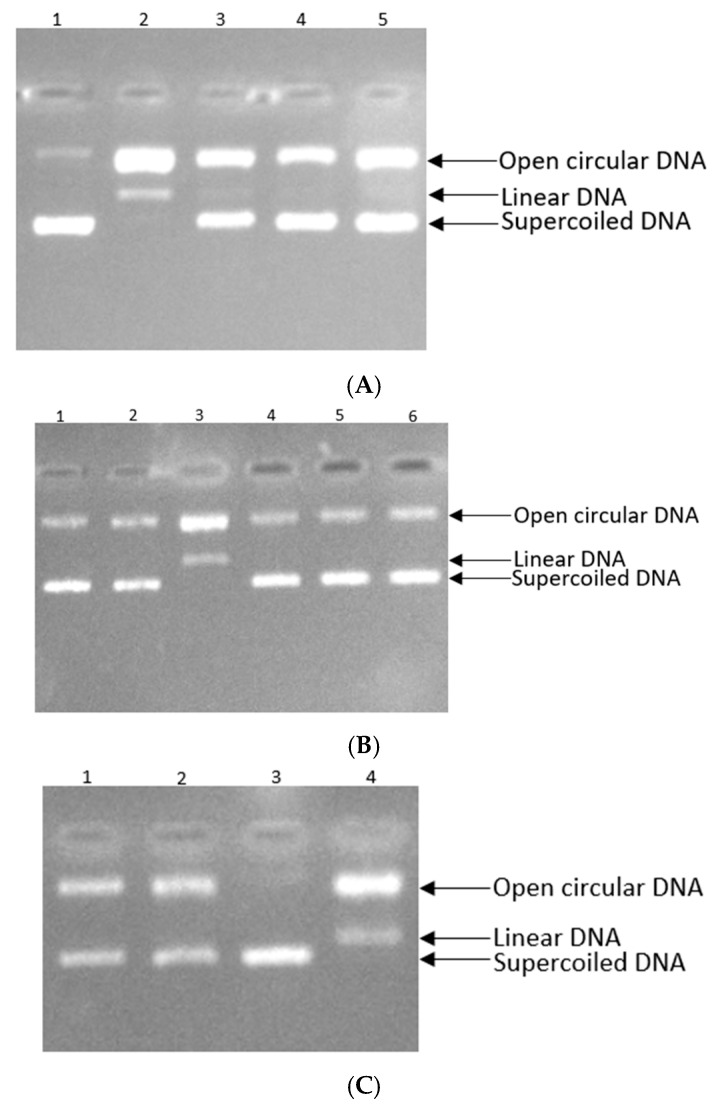
(**A**) Inhibitory effect of natural compounds on pBR322 plasmid DNA damage. Lane 1: control, Lane 2: blank, Lane 3: plasmid DNA + 30 mM AAPH + 8.05 μg/mL (IC_50_) of quercetin, Lane 4: plasmid DNA + 30 mM AAPH + 7.40 μg/mL (IC_50_) of kaempferol, Lane 5: plasmid DNA + 30 mM AAPH + 6.37 μg/mL (IC_50_) of caffeic acid. (**B**) Inhibitory effect of natural compounds on pBR322 plasmid DNA damage. Lane 1: plasmid DNA + 30 mM AAPH + 12.28 μg/mL (IC_50_) of rosmarinic acid, Lane 2: plasmid DNA + 30 mM AAPH + 17.45 μg/mL (IC_50_) of naringenin, Lane 3: blank, Lane 4: control, Lane 5: plasmid DNA + 30 mM AAPH + 9.48 μg/mL (IC_50_) of luteolin, Lane 6: plasmid DNA + 30 mM AAPH + 10.14 μg/mL (IC_50_) of ferulic acid. (**C**) Inhibitory effect of natural compounds on pBR322 plasmid DNA damage. Lane 1: plasmid DNA + 30 mM AAPH + 21.17 μg/mL (IC_50_) of coumaric acid, Lane 2: plasmid DNA + 30 mM AAPH + 21.86 μg/mL (IC_50_) of rutin, Lane 3: control, Lane 4: blank.

The results of this study conclude the effectiveness of the nine polyphenols at protecting against DNA strand scission caused by peroxyl radicals. The protective efficacy of the natural compounds against DNA damage was carried out at concentrations corresponding to their IC_50_ values, as outlined in Section 3.5. The phenolic acid, caffeic acid, demonstrated the highest protective activity against oxidative DNA damage, achieving 98.98% inhibition of DNA strand breakage (Figure 7A, Lane 5). This was followed by the natural flavonol, kaempferol, which achieved 97.34% inhibition (Figure 7A, Lane 4). Furthermore, the flavonol quercetin showed considerable effectiveness in shielding DNA from oxidative damage, inhibiting DNA scission by 96.52% (Figure 7A, Lane 3). Luteolin and ferulic acid also exhibited substantial efficiency in reducing DNA damage (95.87% and 94.17%, respectively), indicating their powerful antioxidant effects (Figure 7B, Lanes 5,6). Moreover, rosmarinic acid and naringenin contribute to the defense mechanisms that counteract free radical-mediated DNA strand breakage, with an inhibition rate of 93.12% and 92.03%, respectively (Figure 7B, Lanes 1,2). The polyphenols, coumaric acid and rutin, showed a weaker antioxidant activity, while they demonstrated a 89.85% and 88.60% inhibition of DNA strand breakage caused by peroxyl radicals (Figure 7C, Lanes 1,2). The values of the strand breakage inhibition rates are in agreement with the values of the antioxidant activity determined by the DPPH method.

## 4. Discussion

The potential of natural compounds for therapeutic development is significant, as evidenced by the growing interest in medicinal plants as a source of bioactive molecules [3,4]. In this study, compounds contained in extracts of *Ocimum basilicum* L., *Mentha piperita* L., *Mentha spicata* L., *and Aloysia citrodora* were evaluated for their potential for antiviral activity against two highly epidemic and clinically significant members of the *Flavivirus* genus, the Dengue Virus (DENV) and the Yellow Fever Virus (YFV).

Natural plant-derived by-products such as polyphenols, commonly found in the daily diet, offer significant benefits to human health, including antioxidant, anti-inflammatory, anticancer, anti-allergic, antihypertensive, and antiviral properties [52]. Plentiful evidence supports the remarkable potential of polyphenols against a broad range of viruses that contribute to widespread health challenges [9]. Indeed, many of these plant-based compounds have demonstrated inhibitory activity against various viruses, including members of the *Flavivirus* genus [22]. Moreover, the close relationship between the antiviral and antioxidant properties of polyphenols suggests that they could contribute to cellular and tissue protection in the human body and regulate the immune response to combat these infections. While polyphenolic compounds have demonstrated antiviral activity against Dengue and Yellow Fever viruses [53,54], their impact on specific stages of the viral life cycle has not been fully elucidated. Previous studies on the phenolic compounds identified as most promising in our work—kaempferol, caffeic acid, rosmarinic acid, and coumaric acid—are limited [55,56,57,58,59], with coumaric acid assessed only through computational approaches to the best of our knowledge. In contrast, our study evaluated these compounds across viral entry, replication, and virion assembly using a quantitative Renilla luciferase reporter system, providing reproducible measurements of viral inhibition. This approach offers mechanistic insight beyond traditional cytopathic effect assays or in silico docking studies, highlighting the direct effects of these compounds on specific stages of the viral life cycle. DENV is a major global health threat with no specific antiviral treatment approved; thus, there is an urgent need for new and effective therapeutic strategies.

In this study, we evaluated nine polyphenolic compounds—ferulic acid, naringenin, quercetin, rosmarinic acid, coumaric acid, rutin, luteolin, caffeic acid, and kaempferol—identified in *Ocimum basilicum* L., *Mentha piperita* L., *Mentha spicata* L., and *Aloysia citrodora* (Table 1, Figure 1), and also common in other Greek medicinal plants [60,61,62]. Our findings revealed that kaempferol and caffeic acid displayed the most pronounced inhibitory effects against DENV genome replication, while coumaric acid was the most effective at blocking viral entry. From the plant extracts analyzed, *Mentha* species contained these three polyphenols, with *Mentha spicata* exhibiting the highest concentration of kaempferol and *Mentha piperita* containing the highest amounts of caffeic acid and coumaric acid. In the case of DENV virus released infectivity, kaempferol once again demonstrated superior activity, exhibiting potent effects at low micromolar concentrations. Specifically, it showed the lowest EC_50_ (3.55 μΜ) and the highest SI (25.45) among all tested compounds and across all stages of the DENV life cycle that we studied. These findings reveal that kaempferol is a particularly promising candidate for further antiviral investigation and development. Apart from kaempferol, caffeic and rosmarinic acid were the only compounds to exhibit inhibitory effects on both viral replication and virus-released infectivity. Again, *Mentha* species and especially *Mentha spicata* exhibit the highest concentration of rosmarinic acid. In the case of YFV, no significant antiviral activity was observed at the viral genome replication stage. This finding highlights a specific mechanism through which these polyphenols exert their inhibitory activity on the DENV life cycle. Our results are consistent with previous studies highlighting the therapeutic promise of polyphenols against DENV [53,55,56,57,58,63]. Specifically, molecular docking analyses have demonstrated kaempferol’s potential to inhibit viral RNA-dependent RNA polymerase (RdRp) by interacting with active site residues, reinforcing its candidacy as an antiviral agent against DENV [55]. Likewise, rosmarinic acid has been reported to inhibit DENV replication by targeting viral enzymes, including NS5 RdRp and nonstructural protein 1 (NS1) [56]. Moreover, rosmarinic acid has been found to bind to envelope domain III (EDIII) across all DENV serotypes (1–4), and plaque assays confirmed its antiviral activity against each serotype [57,58]. Lastly, caffeic acid has been shown to inhibit DENV-1 infection in vitro, as demonstrated by a plaque forming unit (PFU) assay [59].

The selected phytochemical substances were tested concerning their antioxidant activity using the DPPH and ABTS assays, and findings were represented as IC_50_ values. Regarding the DPPH method, our findings demonstrated that all the evaluated compounds exhibited comparable antioxidant activity, as reflected by their IC_50_ value, ranging from 6.37 to 21.86 μg/mL. Despite the differences not being pronounced, some natural compounds exhibited slightly higher radical scavenging activity. Caffeic acid exhibited the greatest level of antioxidant activity, as shown by its lowest IC_50_ value (6.37 μg/mL), while kaempferol (7.40 μg/mL) and quercetin (8.05 μg/mL) showed similar levels of efficacy. These findings are consistent with previous research, which has shown that compounds containing numerous hydroxyl groups and conjugated aromatic systems have high antioxidant potential, enabling effective donation of hydrogen atoms or electrons [64]. Luteolin (9.48 μg/mL) and ferulic acid (10.14 μg/mL) had also substantial antioxidant action, highlighting the effectiveness of hydroxylated flavonoids and phenolic acids [65,66]. Rosmarinic acid, widely renowned for its antioxidant capabilities in medicinal and culinary herbs, also demonstrated significant action (IC_50_ = 12.28 μg/mL). The IC_50_ value of this compound is slightly higher than that of the top-performing compounds; however, it still demonstrates strong free radical scavenging abilities. Similarly, naringenin (IC_50_ = 17.45 μg/mL) significantly contributed to the antioxidant potential and has been validated by literature for its beneficial role in oxidative stress mitigation [67]. Although coumaric acid and rutin had the highest IC_50_ values, they still demonstrated detectable antioxidant activity. Rutin’s lower antioxidant action may be attributed to the presence of sugar moieties, which might limit the availability of free hydroxyl groups required in radical neutralization [68].

The ABTS assay revealed that all nine bioactive compounds exhibit notable radical-scavenging activity, with caffeic acid (IC_50_ = 1.28 μg/mL), kaempferol (IC_50_ = 1.84 μg/mL), and quercetin (IC_50_ = 1.93 μg/mL) displaying the highest antioxidant potency, as indicated by their low IC_50_ values. These findings concur with previous research [69]. The IC_50_ values ranged from 1.28 μg/mL for caffeic acid to 4.54 μg/mL for rutin. This narrow range suggests that the tested polyphenols demonstrate a generally strong and comparable capacity to neutralize ABTS radicals. While the overall antioxidant ranking corresponded with that from the DPPH assay, all bioactive compounds exhibited significantly lower IC_50_ values in the ABTS method. This indicates enhanced scavenging effectiveness against the ABTS radical cation. The observed difference between the DPPH and ABTS methods can be attributed to the greater solubility and reactivity of ABTS radicals, which can be more easily quenched by both hydrophilic and moderately polar antioxidants [70,71]. Therefore, while both assays validated the relative antioxidant capacities of the polyphenols, the ABTS assay consistently showed lower IC_50_ values, highlighting its effectiveness and sensitivity in evaluating the radical scavenging ability of diverse phenolic compounds. Overall, the findings indicate consistent antioxidant activity patterns between the two assays, with the ABTS method providing a more sensitive assessment of the radical scavenging effects.

The findings of the DNA strand breakage inhibition experiment showed that the tested phenolic acids and flavonoids have high antioxidant properties. It was caffeic acid that demonstrated the best inhibition rate, 98.98%, followed closely by the other polyphenols. According to these experimental results, hydroxylated phenolic compounds play a significant role in decreasing oxidative stress through molecular mechanisms [72]. The observed DNA protective trend of the nine polyphenols closely reflects their antioxidant capacity determined by the DPPH and ABTS methods, highlighting a consistent association between radical scavenging activity and protection against DNA damage [73]. Thus, the polyphenolic compounds contained in these plants demonstrate strong antioxidant properties, which may contribute significantly to their antiviral effects by disrupting oxidative stress-dependent pathways involved in viral replication [15].

Our study provides new evidence that polyphenolic compounds contained in Greek medicinal plants have strong antioxidant properties and can interfere with several stages of the DENV life cycle. Most notably, we demonstrate for the first time that these polyphenolic compounds exhibit a significant effect on specific stages of the DENV life cycle. Kaempferol exhibits low-micromolar antiviral activity coupled with a favorable selectivity index, highlighting its potential as a lead compound for future antiviral drug development. These findings reveal the therapeutic potential of plant-derived polyphenols and advance effective, plant-based antiviral strategies. Compared to synthetic drugs, natural compounds offer notable advantages, including broader accessibility, environmental sustainability, and reduced adverse effects, making them promising candidates for future antiviral and antimicrobial interventions.

The identification of pure polyphenolic compounds in the plant extracts provided complementary insights. Although the antioxidant and antiviral assays were performed using commercially available phenolic compounds, the phytochemical analysis of different Greek plant species demonstrates that these substances are naturally present. These plants are commonly consumed as part of the Mediterranean diet, highlighting their dietary and pharmacological relevance. The IC_50_ values determined for the majority of phenolic compounds in the antioxidant assays, as well as the EC_50_ values from the antiviral assays, are consistent with the concentration ranges reported for these compounds in the tested Greek medicinal plant extracts, establishing a stronger link between the observed bioactivities of the individual compounds and their potential contribution to human health.

## 5. Conclusions

Collectively, our findings identify kaempferol and caffeic acid as the most potent compounds against both DENV genome replication and oxidative stress, at concentrations that did not induce cytotoxicity. Since both are present in *Mentha spicata*, this medicinal plant represents a promising candidate for further investigation as a source of antiviral agents. The exploration of plant-derived compounds for antiviral and antioxidant applications offers significant advantages. These natural products are generally more accessible, environmentally sustainable, and less likely to cause adverse effects compared to treatments with synthetic agents [74]. The results of this study underscore the distinctive potential of polyphenols derived from Greek medicinal plants as promising candidates for the development of drugs against DENV. Future research should concentrate on elucidating their detailed mechanisms of action, delivery methods, and possible synergistic effects with current antiviral therapies. Furthermore, it is imperative to conduct in-depth studies on their pharmacokinetics, bioavailability, and safety profiles to facilitate the transition of these promising plant-derived compounds from laboratory discoveries to viable clinical applications.

## Figures and Tables

**Table 1:** Concentration (μg/mL) of the nine polyphenols in *Ocimum basilicum*, *Mentha piperita*, *Mentha spicata,* and *Aloysia citrodora*.

Natural Compound	*Ocimum* *basilicum*	*Mentha piperita*	*Mentha* *spicata*	*Aloysia* *citrodora*
	Concentration (μg/mL)			
(A) Ferulic acid	-	31.92 ± 0.83	-	-
(Β) Naringenin	69.00 ± 1.15	-	-	-
(C) Quercetin	-	6.25 ± 0.46	46.03 ± 0.13	-
(D) Rosmarinic acid	-	6.03 ± 0.52	126.38 ± 0.23	4.83 ± 0.16
(Ε) Coumaric acid	-	32.21 ± 1.02	-	18.66 ± 0.56
(F) Rutin	-	8.32 ± 0.59	40.58 ± 0.09	33.11 ± 0.98
(G) Luteolin	-	27.64 ± 0.32	-	7.79 ± 0.49
(H) Caffeic acid	-	116.89 ± 0.28	19.54 ± 0.56	-
(I) Kaempferol	-	-	7.42 ± 0.45	4.10 ± 0.06

**Table 2:** CC_50_ values of polyphenols obtained after treatment of Huh7 cells for 72 h.

Natural Compound	CC_50_ (μM)
Ferulic acid	>500
Naringenin	373
Quercetin	>500
Rosmarinic acid	>500
Coumaric acid	>500
Rutin	>500
Luteolin	>500
Caffeic acid	>500
Kaempferol	90.36

**Table 3:** EC_50_ and SI values of the nine phenolic compounds for the different stages of the DENV life cycle.

Natural Compound	Replication		Entry + Replication		Infectivity	
	EC_50_ (μM)	SI	EC_50_ (μM)	SI	EC_50_ (μM)	SI
Ferulic acid	>100	-	>100	-	>100	-
Naringenin	>100	-	104.80 ± 4.65	3.55	>100	-
Quercetin	58.46 ± 7.08	8.55	108.70 ± 17.62	4.59	103.90 ± 1.49	4.81
Rosmarinic acid	76.18 ± 7.92	6.56	70.93 ± 9.10	7.04	37.12 ± 0.94	13.46
Coumaric acid	>100	-	47.99 ± 2.43	10.41	91.64 ± 0.40	5.45
Rutin	>100	-	>100	-	>100	-
Luteolin	107.3 ± 1.82	4.65	72.65 ± 7.76	6.88	>100	-
Caffeic acid	35.87 ± 1.88	13.93	>100	-	51.49 ± 1.19	9.71
Kaempferol	31.48 ± 6.39	2.87	35.45 ± 4.09	2.54	3.55 ± 0.07	25.45

**Table 4:** EC50 and SI values for natural compounds against YFV replication.

Natural Compound	EC_50_ (μM)	SI
Ferulic acid	>100	-
Naringenin	101.7 ± 34.37	3.66
Quercetin	>100	-
Rosmarinic acid	98.03 ± 21.03	5.10
Coumaric acid	153.8 ± 29.76	3.25
Rutin	>100	-
Luteolin	>100	-
Caffeic acid	>100	-
Kaempferol	>50	-

**Table 5:** DPPH scavenging activity of nine bioactive compounds.

Bioactive Compounds	IC_50_ Values (μg/mL)
Ferulic acid	10.14
Naringenin	17.45
Quercetin	8.05
Rosmarinic acid	12.28
Coumaric acid	21.17
Rutin	21.86
Luteolin	9.48
Caffeic acid	6.37
Kaempferol	7.40

**Table 6:** ABTS radical scavenging activity of nine bioactive compounds.

Bioactive Compounds	IC_50_ Values (μg/mL)
Ferulic acid	2.51
Naringenin	3.37
Quercetin	1.93
Rosmarinic acid	3.05
Coumaric acid	4.00
Rutin	4.54
Luteolin	2.20
Caffeic acid	1.28
Kaempferol	1.84

## Data Availability

The original data presented in this study are included in the article/Supplementary Materials. For further inquiries, the corresponding author should be contacted.

## Supplementary Materials

The following supporting information can be downloaded at: https://www.mdpi.com/article/10.3390/antiox14091103/s1, Figure S1: Standard calibration curves of the nine bioactive compounds; Table S1: Retention times and UV Absorption Maxima of nine polyphenols; Table S2: CC_50_, EC_50_ and SI values of the nine phenolic compounds for the different stages of the DENV life cycle expresses as μg/mL.
