# Supplementary material for: Evaluation of Polyphenolic Compounds Common in Greek Medicinal Plants for Their Antioxidant Effects and Antiviral Activity Against Dengue and Yellow Fever Viruses
Source: Antioxidants (Basel). 2025 Sep 10;14(9):1103. doi: 10.3390/antiox14091103 (PMC12466706; doi:10.3390/antiox14091103)
Supplement: Supplementary file 1 [file antioxidants-14-01103-s001.zip › antioxidants-3787853-supplementary.pdf]

Supplementary Materials

Evaluation of Polyphenolic Compounds Common in Greek Medicinal Plants for Their Antioxidant Effects and Antiviral Activity Against Dengue and Yellow Fever Viruses

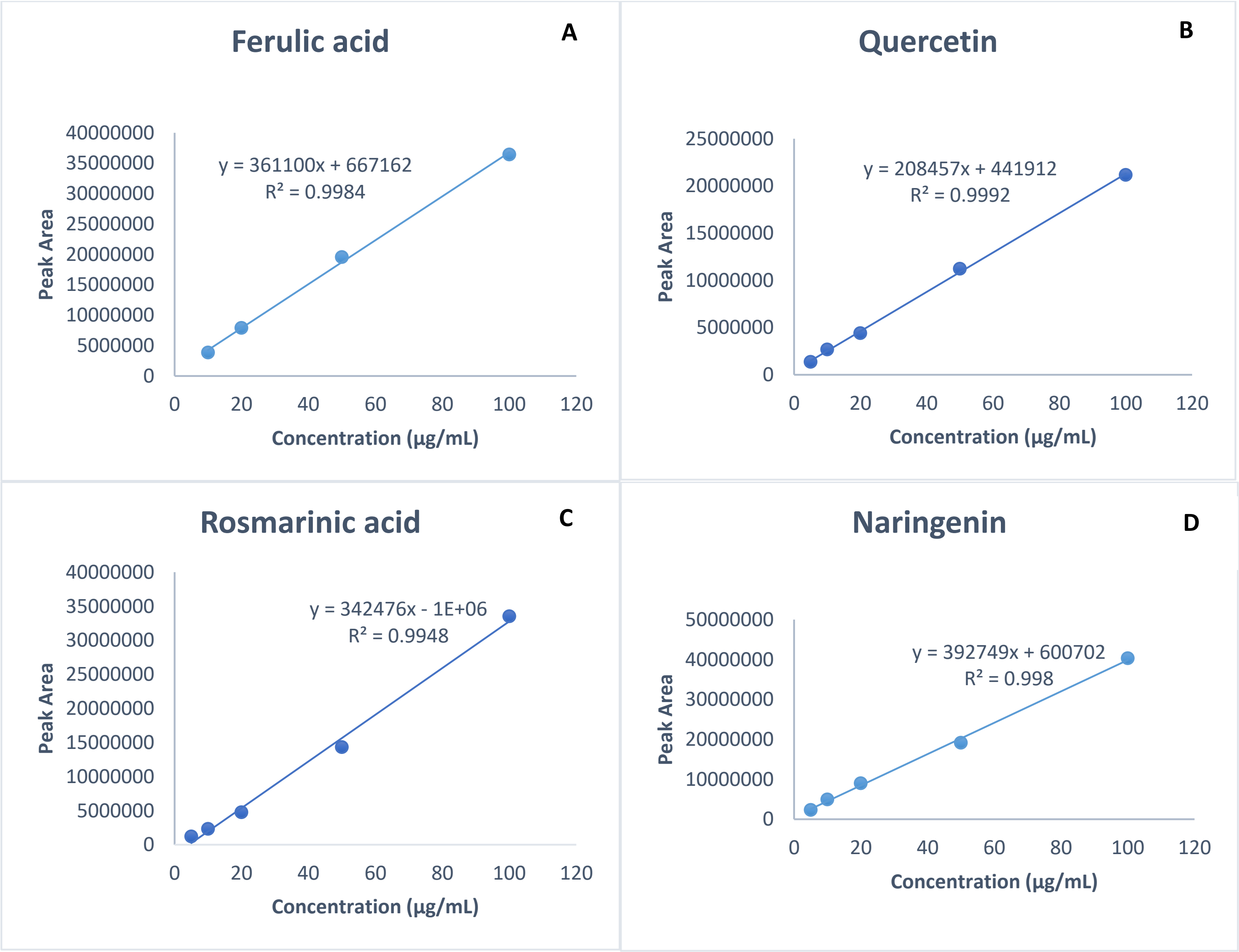

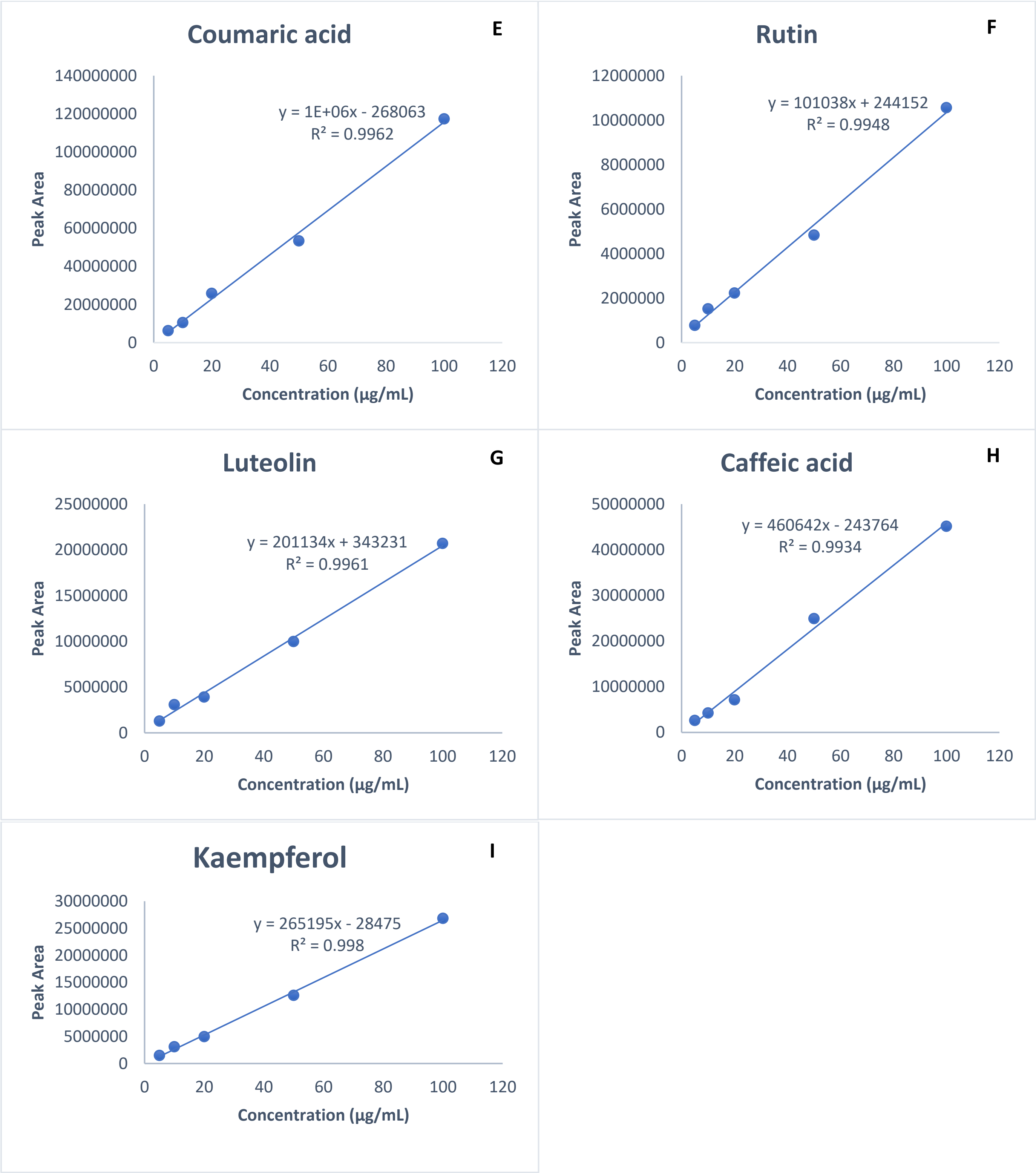

Figure S1. Standard calibration curves of the nine bioactive compounds: A) Ferulic acid, B) Quercetin, C) Rosmarinic acid, D) Naringenin, E) Coumaric acid, F) Rutin, G) Luteolin, H) Caffeic acid and D) Kaempferol.

Table S1. Retention times and UV Absorption Maxima of nine polyphenols.

Bioactive compounds	Retention time (min)	Maximum absorbance wavelength
Ferulic acid	36.77	210, 322
Quercetin	49.24	230, 256, 370
Rosmarinic acid	44.50	206, 245, 330
Naringenin	50.00	235, 289
Coumaric acid	33.50	216, 309, 395
Rutin	40.63	258, 356
Luteolin	49.80	256, 263, 349
Caffeic acid	24.57	209, 240, 323
Kaempferol	52.03	250, 315

Table S2. CC_{50}, EC_{50} and SI values of the nine phenolic compounds for the different stages of the DENV life cycle expresses as μg/ml.

Natural Compound	CC_{50} (μg/ml)	Replication		Entry + Replicaion		Infectivity	
		EC50 (μg/ml)	SI	EC50 (μg/ml)	SI	EC50 (μg/ml)	SI
Ferulic acid	>97.09	>19.41	-	>19.41	-	19.41	-
Naringenin	101.55	>27.22	-	28.53	0.96	27.22	-
Quercetin	>151.12	17.66	2.58	32.12	1.42	31.40	1.45
Rosmarinic acid	>180.15	27.44	2.36	25.55	2.53	13.37	4.84
Coumaric acid	>82.08	>16.41	-	7.87	1.70	15.04	0.89
Rutin	>305.26	>61.05	-	>61.05	-	61.05	-
Luteolin	>143.12	30.71	1.33	20.79	1.96	28.62	-
Caffeic acid	>45.04	3.23	1.25	>4.50	-	4.63	0.87
Kaempferol	>6.466	2.25	0.20	2.53	0.18	0.25	1.82
